# Patient, family and carer experiences of nutritional screening: a systematic review

**DOI:** 10.1111/jhn.12849

**Published:** 2020-12-14

**Authors:** A. F. Bullock, S. L. Greenley, M. J. Patterson, G. A. G. McKenzie, M. J. Johnson

**Affiliations:** ^1^ Wolfson Palliative Care Research Centre Hull York Medical School University of Hull Hull UK; ^2^ Academy of Primary Care Hull York Medical School University of Hull Hull UK

**Keywords:** malnutrition, nutritional screening, patient experience, qualitative, quantitative, systematic review

## Abstract

**Background:**

Despite recommendations for nutritional risk screening of all inpatients, outpatients and care home residents, as well as work to assess clinician's experiences and the validity of tools, little attention has been paid to the experiences of patients undergoing nutritional screening. This review aims to synthesise systematically the current evidence regarding nutritional risk screening with respect to the experiences and views of patients, their families and carers.

**Methods:**

A systematic search was performed in MEDLINE, Embase, PsychINFO, CINAHL, Web of Science and British Nursing Database (inception – July 2019); with screening terms related to malnutrition, screening tools and experience. Titles, abstracts and full‐text papers were independently reviewed by two reviewers and then quality‐appraised. Qualitative papers and quantitative surveys were included. A narrative review of surveys and a thematic framework synthesis of interviews were used to identify themes.

**Results:**

Nine studies, including five qualitative interview papers, were included. Qualitative and quantitative study results were combined using a matrix chart to allow comparison. Surveyed participants reported processes of nutritional screening as acceptable. Three key themes emerged from qualitative data: (i) experience of nutritional screening; (ii) misunderstanding of malnutrition: of causes, role of screening and poor self‐perception of risk; and (iii) barriers to and opportunities for change.

**Conclusions:**

Although the screening process is acceptable, patients’ misunderstanding and poor knowledge regarding causes and consequences of malnutrition result in reduced risk perception and disbelief or disregard of nutritional screening results. Findings should inform policy and clinical practice, as well as highlight the known paucity of data regarding the effectiveness of screening on clinical outcomes.

## Graphical abstract

Despite recommendations for nutritional risk screening, little attention has been paid to the experiences of patients undergoing nutritional screening. This review synthesises the current evidence regarding nutritional risk screening with respect to experiences and views of patients, their families and carers. Conclusions found that although the screening process is acceptable, patients’ misunderstanding of, and poor knowledge regarding causes and consequences of malnutrition result in reduced risk perception and disbelief or disregard of nutritional screening results.

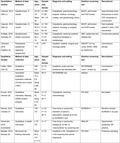

## INTRODUCTION

Screening for the risk of malnutrition is recommended by the National Institute of Health and Care Excellence (NICE) in multiple clinical care settings, including the screening of all hospital inpatients on admission, in addition to hospital outpatients and those in primary care surgeries, both at their first clinic appointment and upon clinical concern, as well as care home residents upon clinical concern.^1^

Given such extensive screening recommendations, validation of screening tools^2^ and their utility and ease of use by clinical staff, including the time taken to complete screening and opinions on the methods, has been conducted.^3^ However, less attention has been paid to the experiences and views of patients, their families and carers when reviewing the acceptability of the screening process. UK National Screening Committee guidance recommends that screening is simple, safe and acceptable to the target population.^4^ Although NICE recommends nutritional screening, the lack of evidence regarding the benefit of screening, or most appropriate way to conduct screening is also highlighted.^1^

Arguments in favour of nutritional screening include early detection and treatment of nutritional problems associated with negative patient outcomes.^5^ However, the impact and the effectiveness of nutritional interventions to manage malnutrition, as a result of heterogeneous and low‐quality studies, remain unclear.^6^, ^7^ Therefore, burdens of screening must be considered alongside any potential benefits because screening may increase anxiety and distress following a positive diagnosis.^8^

This review aims to identify and summarise the available published evidence regarding nutritional screening with respect to the experiences of patients, their families and carers.

## MATERIALS AND METHODS

A systematic review of the literature, including data from both quantitative and qualitative texts, was conducted in accordance with the Cochrane Handbook for Systematic Review of Interventions.^9^ The study protocol was registered with the international prospective register of systematic reviews, PROSPERO (Registration No: CDR42019140859) ^10^ and is reported in accordance with the Preferred Reporting Items for Systematic Review and Meta‐Analyses (PRISMA) guidelines.^11^

### Literature search

Searches were performed by AB and SG on 3 July 2019 in the databases Ovid MEDLINE(R) ALL 1946 to 2 July 2019; Embase via OVID 1974 to Week 26 2019; PsychINFO via OVID 1987 to Week 4 June 2019; CINAHL Complete via EBSCO 1937 to 2 July 2019; ISI Web of Science: Science Citation Index Expanded 1970 to 3 July 2019; and British Nursing Database via ProQuest. The search was updated on 5 June 2020. No limits on publication date or language were applied. The search combined database‐specific indexed terms and textwords related to the two main concepts: Nutritional Assessment of malnutrition, or individual malnutrition screening tools, AND experience or potential harms of screening. The MEDLINE search strategy is outlined in the Supporting information (Material S1), which was translated to alternate databases as required. Forward and backward citation searching of all included studies was completed.

### Inclusion and exclusion criteria

Eligible studies included participants aged 18 years or older, from any clinical setting with any diagnosis. Studies investigating nutritional screening with respect to the views or experiences of patients, their families or informal carers were included. Qualitative and quantitative studies that included surveyed responses or questions regarding views of nutritional screening were included. Studies that reviewed self‐screening of nutritional status, focusing on ‘ease of use’, rather than experiences or opinions of screening, were excluded. Case reports, editorials, opinion pieces and papers reviewing nutritional screening for eating disorders (e.g. anorexia nervosa), were excluded.

### Study selection

All citations retrieved by electronic searching were downloaded to an endnote x8 (https://endnote.com) library, with duplicates removed according to published protocol.^12^ Remaining records were uploaded to covidence systematic review software.^13^ Study titles and abstracts were independently screened (by AB and SG) against eligibility criteria. All potentially relevant studies were retrieved, with full texts reviewed by AB and SG. Disagreements were resolved by consensus or adjudication by a third reviewer (MJ). A custom data extraction form^10^ was used, piloted, reviewed and modified before the final data extraction of included studies was completed (by AB); a random 25% was independently extracted by GM.

### Quality assessment

Each study was appraised using the mixed methods appraisal tool.^14^ All included papers were evaluated by AB with a random 25% being independently reviewed by GM. Disagreements were resolved by consensus. For quality assessment of studies, see the Supporting information (Material S2).

### Analysis

A narrative summary with descriptions and comparisons was completed for quantitative studies, providing an initial descriptive summary and explanation of characteristics of the included studies.^15^, ^16^ A narrative approach was used to analyse the relationship within and between studies, and assess the overall strength of the evidence.^15^ Qualitative results were reported in accordance with the Enhancing Transparency in Reporting the Synthesis of Qualitative research (ENTREQ) guidance.^17^ Thematic synthesis was used for the qualitative findings using Thomas and Harden methodology.^18^ Combining qualitative findings allowed new and generalisable knowledge to be generated. Synthesis was performed in three stages: (i) initial data coded regarding experiences of nutritional screening (conducted by AB); (ii) descriptive themes generated, with codes grouped into categories (AB and MP); and (iii) analytical themes generated both inductively and deductively, with the investigators (AB and MP) generating themes independently, then through discussion with a third investigator (MJ). Participants quotes and the interpretations of responses by the authors of the studies were used within the qualitative synthesis. Results from qualitative and quantitative syntheses were combined and charted into a matrix to allow final comparison between studies (see Supporting information, Material S3). In view of the focussed nature of the synthesis, a theoretical framework was not used to underpin the analysis.

## RESULTS

**Figure 1:**
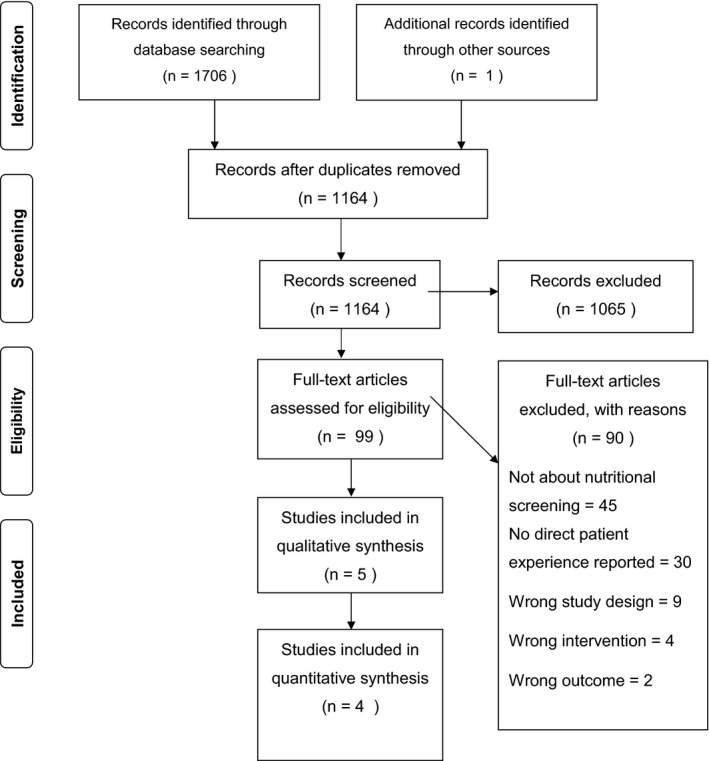
PRISMA flow diagram.

Searches returned 1164 unique articles after deduplication, with 99 studies included for full‐text screening. From this, nine studies published between 2004 and 2019 were eligible for inclusion, representing 609 participants, including 83 participants from five qualitative studies (see PRISMA flow chart, Figure 1).

### Design, sample size and setting

Table 1 provides a summary description of the included studies. Three studies used questionnaires,^19^, ^20^, ^21^ one of which^21^ included free‐text comments. A fourth comprised researchers’ opinions of patients’ views.^22^

**Table 1:** Study characteristics summary

	Method of data collection	Age (years)	Sample size (gender)	Diagnosis and setting	Nutrition screening tool	Recruitment
*Quantitative studies*						
Cawood *et al*. (2012) USA^19^	Questionnaire, % results	Mean 55 years, range 18–87 years,	*n* = 205 F = 90 M = 115	Outpatients; gastroenterology, surgical, medical, oncology, urology, and gynaecology clinics	MUST; self‐screen and HCP screen	Approximately every third person in clinic; 72% consented to involvement
Cawood *et al*. (2018) USA^20^	Questionnaire, % results	Mean (SD) 50.4 (16.2) years	*n* = 100 F = 43 M = 57	Outpatients; gastroenterology, medical, oncology or surgical clinics	MUST; self‐screen and HCP screen	Next available patient in clinic, HCP recruitment
Di Bella *et al*. (2018) Australia^21^	Questionnaire, written comments	Mean (SD) 58 (16) years	*n* = 160 F = 67 M = 93	Outpatients; receiving systemic supportive therapies or radiotherapy	MST; patient‐led and dietitian‐led	Consecutive patients
Tammam *et al*. (2019) England^22^	Participants questioned regarding assessment	>18 years	*n* = 61	Inpatient; medical, surgical and oncology wards	INSYST I & II by nurse, MUST, MNA by researcher	Convenience sample
*Qualitative studies*						
Callen (2004) USA^23^	Qualitative interviews, naturalistic qualitative evaluation methods of Guba and Lincoln (1981)	≥65 years, mean (SD) 74 (6.6) years, range 68–86 years	*n* = 10 F = 4 M = 6	Inpatients; acute services. Nutritional risk identified with DETERMINE tool	DETERMINE Level 1 screen by dietitian	Convenience sample
Kroner *et al*. (2012) German^24^	Qualitative interviews, Mayring, (2008) content analysis	Mean 63 years, range 37–84 years	*n* = 12 F = 5 M = 7	Outpatients, receiving chemotherapy	PG‐SGA	Not stated
Reimer *et al*. (2012) Canada^25^	Qualitative interviews	>55 years	*n* = 22 F = 13 M = 9	Free‐living in community, members of senior's association; classed as at risk by SCREEN II tool	SCREEN II	Random sample; SCREEN II via post
Hamirudin, 2016 Australia^26^	Qualitative, in‐depth interviews	≥75 years	*n* = 17	Free‐living in community; classed as ‘at risk’ or ‘malnourished’ by screening tool	MNA‐SF	Opportunistic screening; GP practice
Balstad *et al*. (2019) Norway^27^	Structured de‐briefing interviews	Mean (SD) 64.4 (11.9) years	*n* = 23	Inpatients *n* = 22, Outpatient *n* = 1, *n* = 22 receiving anti‐cancer treatments	PG‐SGA	Purposive sampling

DETERMINE, DETERMINE Your Nutritional Health; HCP, Health Care Professional; INSYST I & II, Imperial Nutritional Screening System I & II; MNA (Mini Nutritional Assessment); MNA‐SF, Mini Nutritional Assessment Short Form; MST, Malnutrition Screening Tool; MUST, Malnutrition Universal Screening Tool; PG‐SGA, Patient Generated Subjective Global Assessment; SCREEN II, Seniors in the Community – Risk Evaluation for Eating and Nutrition; M, male; F, female.

Five studies were of qualitative interviews.^23^, ^24^, ^25^, ^26^, ^27^ Sample sizes ranged from 61^22^ to 205^19^ for quantitative studies and from 10^23^ to 23^27^ for qualitative studies. Four studies were conducted in outpatient settings,^19^, ^20^, ^21^, ^24^ three in inpatient settings^22^, ^23^, ^27^ and two in the community.^25^, ^26^ Studies were conducted in the USA,^19^, ^20^, ^23^ Canada,^25^ Australia,^21^, ^26^ Germany,^24^ Norway^27^ and England.^22^

### Participants

Participants with a range of medical conditions were represented, including those receiving medical or surgical treatments,^19^, ^20^, ^22^, ^24^, ^27^ including anticancer treatments, such as chemotherapy and radiotherapy,^21^, ^24^, ^27^ and free‐living individuals without significant morbidity.^25^, ^26^ Various recruitment methods were used, including consecutive^20^, ^21^ and sequential^19^ inclusion of clinic patients, and convenience^22^ sampling of inpatients in quantitative studies. Qualitative studies used convenience,^23^ random^25^ opportunistic^26^ or purposive^27^ sampling. One study did not state recruitment methods.^24^ No papers were identified that captured experiences of patient's families or informal carers.

Various malnutrition screening tools were used: Malnutrition Universal Screening Tool (MUST)^19^, ^20^, ^22^; Malnutrition Screening Tool (MST)^21^; Imperial Nutritional Screening System I and II Tools (INSYST I & II)^22^; Mini Nutritional Assessment (MNA)^22^; DETERMINE Your Nutritional Health (DETERMINE) checklist^23^; Patient Generated Subjective Global Assessment (PG‐SGA)^24^, ^27^; Seniors in the community – Risk Evaluation for Eating and Nutrition II tool (SCREEN II)^25^; and the Mini Nutritional Assessment – Short Form (MNA‐SF)^26^ (Table 1).

### Questionnaire findings

Three studies^19^, ^20^, ^21^ collected data regarding participant's experiences of screening using questionnaires. The fourth^22^ evaluated the acceptability of the tool by asking participants their subjective opinions regarding the tool. From these, most participants reported that they were agreeable towards nutritional screening, with 99%^19^ and 100%^20^ of participants in two studies reporting they were happy to answer questions regarding their nutrition. Written comments^21^ included three positive responses of screening as a ‘good idea’ and four negative comments, suggesting nutritional screening was ‘unnecessary’. Requests for explanation of screening results were made.^19^ Finally, the fourth study^22^ where comments from participants had been noted suggested that most were comfortable with the screening process and recognised the importance of screening.

### Interview findings

Three key themes emerged: (i) experience of nutritional screening; (ii) misunderstanding of malnutrition; and (iii) barriers to and opportunities for change.

#### Experience of nutritional screening

Comments regarding screening tool content or process were common, with data generating a theme regarding the acceptability of being screened. Participants found screening to be simple,^23^, ^26^ and possible as part of a routine assessment^25^ Questions asked were acceptable, and participants did not feel they were too sensitive or intrusive.^24^, ^26^

‘Well it’s quite simple. When you get to my age, you want things simple don’t you?’^26^

However, some participants were unclear on what had been examined, or of the purpose of nutritional screening.^24^ Completion of questionnaires also caused some participants distress, particularly when discussing unintentional weight loss, or negative changes to their physical condition.^27^

‘I want to avoid this! [refers to question about weight loss]. The hardest thing is when you lose weight when you actually don’t want to’^27^

#### Misunderstanding of ‘malnutrition’

A key theme was seen regarding participants misunderstanding of the term malnutrition, with many believing that ‘malnutrition’ was not following a ‘healthy diet’, high in fruits, vegetables and wholegrains, or that being overweight precluded malnutrition.^23^, ^24^, ^25^

‘I’m 280 pounds. How can I be malnourished?’^23^

This requirement to follow a ‘healthy diet’ was reinforced by the media (e.g. magazines), and family members, if participants had received a new medical diagnosis (e.g. cancer).^24^, ^26^ Participants had a poor understanding of malnutrition and its contributory factors; with participants reporting that their overall nutritional health was ‘fair’ or ‘good’, even if screening showed a nutritional issue to address.^23^

‘Well I couldn’t understand that. When I eat properly – I feel I eat properly – I couldn’t understand why … then it showed I was malnourished’^26^

As a result of this misunderstanding, some participants reacted negatively when informed of their nutritional risk, and were disappointed or upset with screening results.^25^, ^26^ Some felt accused of having an inadequate diet,^25^ or having a poor knowledge of nutrition when they believed they were well‐informed.^23^

‘I was initially kind of shocked that I scored … you know’^25^

So in what way do you feel I … I’m not doing the right things?’^25^

This caused participants to justify their current dietary intake, and describe how they had cut down on ‘bad’ foods and were making an effort to consume the ‘right’ foods, including changing snacks to fruit, consuming wholegrain foods, or reducing red meat intakes.^23^, ^24^, ^25^, ^26^

‘Yeh well I eat loads of vegetables and so I found it ah … I am doing things right’^25^

‘Now I eat fruit instead of chocolate’^24^

##### Risk perception

Further misunderstandings of malnutrition's causes and consequences were seen in participants who had lost weight. Participants saw weight loss as a positive, as a result of previously being overweight,^24^ and rationalised weight loss as the result of healthy dietary changes, rather than their diagnosis. Weight loss was also seen as a normal part of ageing,^26^ and was not associated with disease.^24^

‘Yes, I noted it [weight loss], I’m better off, I was a bit too snug’^24^

However, some participants credited weight loss as a cause of physical weakness, and saw weight loss as a negative event.^24^, ^27^

‘I have lost a lot of weight, seven kilos, it was the end of my strength. It [weight loss] was bad and depressing’^24^

As a result of beliefs that being overweight or following a ‘healthy’ diet precluded malnutrition, participants did not see themselves as ‘at risk’. With this, nutritional screening results were not prioritised, and advice to manage malnutrition was declined or ignored.^25^, ^26^ Participants also compared their own risk to others, feeling their risk was comparatively low; this was supported by a perceived lack of symptoms related to malnutrition.^25^

‘I don’t feel I’m as much at risk as … as the community at large. And that’s what bothers me are the people out there. They’re far more at risk I feel’^25^

Symptoms, such as weight loss, were seen as a normal part of ageing, or the disease process (e.g. cancer), and therefore were not seen as modifiable^24^, ^26^

‘Well they can't do much. It's me getting old, tired and worried and well, you know’^26^

##### Results of screening

Reactions to results of screening varied. On reviewing results, rather than focusing on nutritional risk, participants noted positive aspects of their current diet.^25^, ^26^ A focus on ‘room for improvement’ was seen; with screening results seen as affirmation of aspects of their diet they were getting ‘right’ rather than highlighting areas which required intervention.^23^, ^25^ Similarly, participants often dismissed results or advice, as weight loss was attributed to other perceived unrelated factors, such as cancer therapies, or a belief that their current knowledge or actions were sufficient.^24^, ^26^

‘I don't need it. No, we look after ourselves as far as cooking and eating is concerned. I think common sense has got a lot to do with it’^26^

Interpretation of nutritional risk was also contextualised in light of other health concerns or social situations,^23^, ^24^, ^25^ particularly if participants felt they were eating well,^23^, ^26^ therefore dietary changes were not a priority.

‘Well because of the issues I have with my son and his children, I didn't really take an awful lot of notice of it I'm afraid. I’m sorry, I should have but I didn't’^26^

#### Barriers to and opportunities for change

##### Barriers to change, misinformation and rejection

Several barriers to changing dietary intake emerged. Results of screening were dismissed as irrelevant, incorrect or unrequired^24^, ^25^, ^26^ if participants felt they were eating well, or were consuming a ‘healthy’ diet, and resulted in participants declining information aimed at improving their nutritional status.^26^

'Well I couldn’t understand that. When I eat properly – I feel I eat properly – I couldn’t understand why … then it showed I was malnourished'^26^

Poor appetite, caused by ageing or diseases status, was as barrier to change.^23^, ^24^, ^25^ Similarly, social circumstances and lifetime habits, such as cooking and food choices, also presented as barriers, meaning that nutritional information was not prioritised above other concerns or habits.^25^, ^26^

Nutritional recommendations were also rejected because of participants feeling information provided was not personalised, and the methods and results of mass nutritional screening were not applicable to themselves as individuals.

‘The recommendations were good for the average person, but like I said, I believe that I eat and watch my diet quite well’^25^

##### Opportunity for change

Conversely, some participants were pleased the topic of nutrition was addressed, and felt they may benefit from nutritional recommendations.^24^, ^25^, ^26^ However, this was often seen as ‘room for improvement’,^23^, ^25^ rather than a requirement to change.

‘I count on the medical profession to let me know if they see that there is something wrong. If my weight drops or whatever, then I hope they will ring bells and say “Hey!”’^25^

## DISCUSSION

We provide the first systematic review and synthesis of nutritional screening with respect to the experiences of patients, their families and carers. The results of this review suggest that participants found nutritional screening to be acceptable. Despite this, issues regarding the relevance, understanding and value of nutritional screening must be noted. Reaction to the results of screening was mixed, and included disbelief, disappointment and offence, as well as being seen by some as an opportunity for learning. Poor understanding of malnutrition, misattribution of risk and perceived barriers contributed to low prioritisation and indifference to the results and nutritional advice given.

Although the survey responses suggest nutritional screening is perceived as an acceptable process, and completion of screening tools themselves was not burdensome, analysis of qualitative papers regarding the usefulness and applicability of nutritional screening raise questions regarding the effectiveness of nutritional screenings.

The qualitative and survey responses align regarding the acceptability of the screening process; however, some participants did not understand the purpose of screening, or what was being screened for. Similarly, results showing the risk of malnutrition were met with disbelief or indifference because malnutrition and the role of screening were not well understood, and therefore not prioritised. This lack of understanding of malnutrition and its role in ageing, disease and overall health, meant that participants expressed little concern regarding a diagnosis of malnutrition risk; with perceptions of good nutrition focused on following a ‘healthy’ diet, rather than one appropriate for their current medical condition. Importantly, generic nutrition support advice was often rejected because participants perceived themselves to either require individualised advice (e.g. as a result of comorbidities, or not seeing themselves as one of the majority).

Common barriers to change included incorrect assumptions that weight loss and poor appetite were a normal part of ageing, or an expected part of disease. A recent systematic review^28^ identifying barriers and facilitators to nutritional screening in the community, which included both patient and HCP responses, identified similar barriers, including reluctance to be screened, lack of recognition of malnutrition and its importance, and avoidance of ‘unhealthy’ calorie‐dense foods. Moreover, our review suggested that perceptions regarding the positives of weight loss and avoidance of ‘unhealthy’ foods were reinforced by family and media encouragement to follow a ‘healthy’ diet.

Mass nutritional screening is recommended as per NICE^1^; however, its benefit has yet to be demonstrated. A Cochrane review examining the effectiveness of nutritional screening on patient outcomes and quality of care found that there was insufficient evidence in the support of screening, although no evidence of ineffectiveness was found.^29^ Similarly, NICE guidance recommending nutritional screening is solely based upon expert clinical opinion, and the effectiveness of nutrition support to manage malnutrition risk is unclear because previous studies demonstrated little overall effect on mortality, and carried a high risk of bias.^1^, ^6^, ^30^ Considerations of the cost‐effectiveness and validity of methods of screening are also required when appraising the appropriateness and viability of screening methods, and include the condition being screen for showing benefit of treatment, and the benefits weighted against possible harms cause by screening (e.g. anxiety, overdiagnosis).^4^, ^31^

Concerns regarding the harm of screening are more often considered when discussing screening for diseases such as cancer, where the harm of testing procedures, diagnostic false‐positives and anxiety caused by screening itself is more tangible.^8^, ^30^ However, the potential harm of nutritional screening, as identified by this review, includes the distress of being informed of results, particularly if participants felt they were following a ‘healthy’ diet, or the screening tool highlighting negative physical attributes (e.g. significant weight loss). This may cause resistance to change, or reluctance to accept advice to manage nutritional risk. With the lack of evidence regarding the role and benefit of screening, as well as the results of this review suggesting that screening results are poorly understood, questions regarding the effectiveness of nutritional screening, when the public understanding of the condition is poor, must be considered.

### Implications for clinical practice, research and policy

This review identified several areas which require further considerations when implementing nutritional screening programmes. Foremost, knowledge regarding malnutrition, both its causes and consequences, must be addressed to allow informed interpretation of screening results. Primarily, misconceptions that weight loss is always a positive health outcome, and that consumption of calorie‐dense foods is always ‘unhealthy’, must be addressed.

Education for vulnerable groups regarding the role of nutritional screening, malnutrition, and its causes and consequences, combined with a tailored approach to providing nutritional advice, may help support behaviour change, particularly in societies where key public health messages are aimed at combatting obesity.

With this, further research regarding the most appropriate and effective interventions to identify and manage malnutrition should be conducted to prevent psychological or physical distress when there is no prospect of benefit (e.g. anxiety or disbelief of results resulting in disengagement) or provision of inappropriate treatments (e.g. for patients with refractory cachexia).^32^

How to alter public health messages, aiming to encompass requirements for different nutritional needs across the lifetime, as well as between the two public health considerations of obesity and malnutrition, also requires consideration.

### Strengths and limitations

The use of a mixed methods design is a main strength of this review, with both qualitative and quantitative studies being included in the analysis. This allowed the triangulation of results and enabled a richer insight into patients’ experiences of nutritional screening.

Although this review only included nine studies, the depth of information gained from the five included qualitative studies (which included 83 participants) regarding the specific topic of nutritional screening provides a robust assessment of patients’ views of nutritional screening.^33^ However, as a result of the limitations identified in the original articles, including some limited sample sizes, as well as a lack of diversity in research populations, caution is required when interpreting results, and further research regarding patients’ experiences of nutritional screening is required.

Additionally, we did not use a theoretical framework underpinning the qualitative analysis. However because of the narrow topic and the small number of studies included, the absence of a framework is unlikely to have weakened the results.

The studies included in this review were from high income countries, where issues of obesity, its associated comorbidities and the requirement for weight loss to manage these conditions together comprise a key public health message. Therefore, the generalisability of some findings (e.g. weight loss seen as positive) may be limited to societies where obesity is considered to be a greater concern than malnutrition.

## CONCLUSIONS

Misunderstanding, caused by a lack of knowledge regarding the causes and consequences of malnutrition, resulted in reduced risk perception and disbelief or the rejection of screening results. Nutritional screening can be a trigger for dietary changes, although barriers, including older age, lifetime habits, disease status and social factors, particularly family and media encouragement of ‘healthy’ diets, meant that nutritional problems were not prioritised, particularly when weight loss and poorer dietary intake were seen as a normal part of ageing and the disease process. This resulted in low prioritisation of screening results and associated recommendations. The effectiveness and appropriateness of nutritional screening, when results are misunderstood and risk is misattributed to disease or ageing, must be considered, particularly when the efficacy of nutritional interventions to manage malnutrition is unknown. Although the process of screening is acceptable, without addressing patient barriers, particularly a fundamental lack of knowledge regarding malnutrition, in the context of a paucity of cost‐effectiveness data, the role of nutritional screening must be questioned.

## Conflict of interests, source of funding and authorship

The authors declare that they have no conflicts of interest.

AB and MJ designed the project. AB, SG and MJ designed the protocol. AB and SG conducted the review. AB and GM performed the extraction of data. AB and MP performed the analysis. All authors revised the manuscript critically. AB and MJ had overall responsibility for the final content.

## Transparency declaration

The authors affirm that this manuscript is an honest, accurate and transparent account of the study being reported. The authors affirm that no important aspects of the study have been omitted and that any discrepancies from the study as planned have been explained.

## Supporting information

Material S1. MEDLINE search strategy.

Material S2. Study quality assessments.

Material S3. Charted matrix to allow comparison between studies.
