# Supplementary material for: Patient, family and carer experiences of nutritional screening: a systematic review
Source: J Hum Nutr Diet. 2020 Dec 14;34(3):595–603. doi: 10.1111/jhn.12849 (PMC8246934; doi:10.1111/jhn.12849)
Supplement: Supplementary file 1 — Material S1. MEDLINE search strategy. [file JHN-34-595-s001.docx]

**Online Supplementary Material One**MEDLINE Search

| 1. Nutrition Assessment/ |  |
| --- | --- |
| 2. ((malnutrition* or nutrition*) adj3 (screen* or risk*)).ti,ab. |  |
| 3. Mass Screening/ |  |
| 4. (malnutrition* or nutrition*).ti,ab. |  |
| 5. Nutritional Status/ |  |
| 6. *Malnutrition/di |  |
| 7. Stress, Psychological/ |  |
| 8. overdiagnosis.ti,ab. |  |
| 9. Anxiety/ |  |
| 10. false negative reactions/ or false positive reactions/ or (false adj (negative or positive)).ti,ab,kw. |  |
| 11. exp Pain/ |  |
| 12. Unnecessary Procedures/ |  |
| 13. Psychological Trauma/ |  |
| 14. Patient Satisfaction/ |  |
| 15. "Patient Acceptance of Health Care"/ |  |
| 16. ((user* or people* or patient* or consumer* or adult* or subject* or caregiver* or care giver* or family or families or spouse* or relative* or carer*) adj4 (feeling* or emotion* or view* or experience* or perception* or perspective* or opinion* or accept* or satisfaction)).ti,ab,kw. |  |
| 17. ((harm* or adverse*) adj4 screen*).ti,ab. |  |
| 18. 7 or 8 or 9 or 10 or 11 or 12 or 13 or 14 or 15 or 16 or 17 |  |
| 19. 4 or 5 |  |
| 20. 3 and 19 |  |
| 21. 1 or 2 or 20 |  |
| 22. 18 and 21 |  |
| 23. 6 and 18 |  |
| 24. 22 or 23 |  |
| 25. limit 24 to ("all infant (birth to 23 months)" or "all child (0 to 18 years)" or "newborn infant (birth to 1 month)" or "infant (1 to 23 months)" or "preschool child (2 to 5 years)" or "child (6 to 12 years)" or "adolescent (13 to 18 years)") |  |
| 26. limit 24 to ("all adult (19 plus years)" or "young adult (19 to 24 years)" or "adult (19 to 44 years)" or "young adult and adult (19-24 and 19-44)" or "middle age (45 to 64 years)" or "middle aged (45 plus years)" or "all aged (65 and over)" or "aged (80 and over)") |  |
| 27. 25 not 26 |  |
| 28. 24 not 27 |  |
| 29. BAPEN.ti,ab,kw. |  |
| 30. "British association for parenteral and enteral nutrition".ti,ab,kw. |  |
| 31. BNST.ti,ab,kw. |  |
| 32. "British Nutrition* Screening Tool".ti,ab,kw. |  |
| 33. CNST.ti,ab,kw. |  |
| 34. "Canadian Nutrition* Screening Tool".ti,ab,kw. |  |
| 35. CONUT.ti,ab,kw. |  |
| 36. "Controlling Nutrition* Status".ti,ab,kw. |  |
| 37. ESPEN diagnostic criteria for malnutrition.ti,ab,kw. |  |
| 38. (EDC and malnutrition).ti,ab,kw. |  |
| 39. GNRI.ti,ab,kw. |  |
| 40. Geriatric Nutrition* Risk Index.ti,ab,kw. |  |
| 41. INSYST.ti,ab,kw. |  |
| 42. Imperial Nutritional Screening System.ti,ab,kw. |  |
| 43. "Imperial Nutrition and Metabolism".ti,ab,kw. |  |
| 44. MST.ti,ab,kw. |  |
| 45. Malnutrition screening tool.ti,ab,kw. |  |
| 46. MSTC.ti,ab,kw. |  |
| 47. Malnutrition Screening Tool for Cancer.ti,ab,kw. |  |
| 48. Malnutrition Universal Screening Tool.ti,ab,kw. |  |
| 49. (MUST and malnutrition).ti,ab,kw. |  |
| 50. Nutrition* risk index.ti,ab,kw. |  |
| 51. NRI.ti,ab,kw. |  |
| 52. ((NRS-2002 or NRS) adj "2002").ti,ab,kw. |  |
| 53. Nutrition* Risk Screening.ti,ab,kw. |  |
| 54. NUFFE.ti,ab,kw. |  |
| 55. Nutrition* form for the elderly.ti,ab,kw. |  |
| 56. SGA.ti,ab,kw. |  |
| 57. ((PG-SGA or PGSGA or PG) adj SGA).ti,ab,kw. |  |
| 58. Subjective global assessment.ti,ab,kw. |  |
| 59. Patient Generated Subjective Global Assessment.ti,ab,kw. |  |
| 60. SNAQ.ti,ab,kw. |  |
| 61. ((simplified or short) adj nutrition* assessment questionnaire).ti,ab,kw. |  |
| 62. 3 Minute Nutrition* Screening.ti,ab,kw. |  |
| 63. 3-MinNS.ti,ab,kw. |  |
| 64. PNI.ti,ab,kw. |  |
| 65. prognostic nutrition* index.ti,ab,kw. |  |
| 66. MNA.ti,ab,kw. |  |
| 67. mini nutrition* assessment.ti,ab,kw. |  |
| 68. MNA-SF.ti,ab,kw. |  |
| 69. or/29-68 |  |
| 70. 4 and 69 |  |
| 71. 18 and 70 |  |
| 72. limit 71 to "all child (0 to 18 years)" |  |
| 73. limit 71 to "all adult (19 plus years)" |  |
| 74. 72 not 73 |  |
| 75. 71 not 74 |  |
| 76. 28 or 75 |  |
| 77. (animals not humans).sh. |  |
| 78. 76 not 77 |  |
