# Supplementary material for: Patient, family and carer experiences of nutritional screening: a systematic review
Source: J Hum Nutr Diet. 2020 Dec 14;34(3):595–603. doi: 10.1111/jhn.12849 (PMC8246934; doi:10.1111/jhn.12849)
Supplement: Supplementary file 2 — Material S2. Study quality assessments. [file JHN-34-595-s002.docx]

**Online Supplementary Material Two: study quality assessment**

Mixed Methods Appraisal Tool (MMAT) Quality assessment

|  | | **4. QUANTITATIVE DESCRIPTIVE STUDIES** | | | | | **1. QUALITATIVE STUDIES** | | | | |
| --- | --- | --- | --- | --- | --- | --- | --- | --- | --- | --- | --- |
| First author, year | | 4.1. Is the sampling strategy relevant to address the research question? | 4.2. Is the sample representative of the target population? | 4.3. Are the measurements appropriate? | 4.4. Is the risk of nonresponse bias low? | 4.5. Is the statistical analysis appropriate to answer the research question? | 1.1. Is the qualitative approach appropriate to answer the research question? | 1.2. Are the qualitative data collection methods adequate to address the research question? | 1.3. Are the findings adequately derived from the data? | 1.4. Is the interpretation of results sufficiently substantiated by data? | 1.5. Is there coherence between qualitative data sources, collection, analysis and interpretation? |
| Callen | 2004 |  |  |  |  |  | Yes | Yes | Can't tell | No | Yes |
| Balstad | 2019 | Yes | Can’t tell | Yes | No | Yes |  |  |  |  |  |
| Cawood | 2012 | Yes | Can't tell | Can't tell | No | Yes |  |  |  |  |  |
| Cawood | 2018 | Yes | Can't tell | Yes | No | Yes |  |  |  |  |  |
| Di Bella | 2018 | Yes | Can't tell | Yes | Yes | Yes |  |  |  |  |  |
| Hamirudin | 2016 |  |  |  |  |  | Yes | Yes | Yes | Yes | Yes |
| Kroner | 2012 |  |  |  |  |  | Yes | Yes | Yes | Yes | Yes |
| Reimer | 2012 |  |  |  |  |  | Yes | Yes | Yes | Yes | Yes |
| Tammam | 2009 | No | Can't tell | Yes | Can't tell | Yes |  |  |  |  |  |
