# Supplementary material for: Patient, family and carer experiences of nutritional screening: a systematic review
Source: J Hum Nutr Diet. 2020 Dec 14;34(3):595–603. doi: 10.1111/jhn.12849 (PMC8246934; doi:10.1111/jhn.12849)
Supplement: Supplementary file 3 — Material S3. Charted matrix to allow comparison between studies. [file JHN-34-595-s003.docx]

**Online Supplementary Material Three**

Charted matrix to allow comparison between studies

| **Theme** | **Sub-theme** | **Qualitative articles, quotes** | **Quantitative articles, results** |
| --- | --- | --- | --- |
| **Theme 1**: Experience of screening | -- | “That doesn’t worry me on iota” (F7) ^(26)^  “Well it’s quite simple. When you get to my age, you want things simple don’t you?” (M1) ^(26)^  “Yes, it was very good that the topic [nutrition] was addressed” (TN09) ^(24)^  Participants were not clear on what was examined “Yes, what did she do?” (TN 09) ^(24)^  Did not feel the assessment was unpleasant or disturbing ^(24)^  “Oh my God, I want to avoid this! [refers to question about weight loss]. The hardest thing is when you lose weight when you actually don’t want to” (P13, Woman) ^(27)^  When the form asked about functional decline and weight loss, it was difficult for some participants to answer honesty “I wish I could have selected “able to do little activity and spend most of the day in bed or chair”. But to be honest, I have been lying in bed”. (Participant 16, man). | 99% of participants were happy to answer questions regarding their nutrition ^(19)^  100% participants were happy to answer questions about their nutrition ^(20)^  Participants found the screening process acceptable ^(21)^  Questions were easy to understand (MST tool) ^(21)^  Screening may be confusing, or unnecessary ^(21)^  Participants were comfortable with screening process ^(22)^ |
| **Theme 2**: Understanding of malnutrition | Misunderstanding of malnutrition  (not understood therefore following ‘healthy eating’ advice)  (disbelief of results as following heathy eating advice) | “Yes, I noticed it [weight loss], I’m better off, I’m was a bit too snug” (TN 01) ^(24)^  “I have lost a lot of weight, seven kilos, it was the end of my strength. It [weight loss] was bad and depressing” (TN 07) ^(24)^  Information gathered from magazines and family members: encouraged ‘healthier’ diets ^(24)^  “Then I drank actimel instead of water” (TN 08) ^(24)^  “Well I couldn’t understand that. When I eat properly – I feel I eat properly – I couldn’t understand why… then it showed that I was malnourished” (F5) ^(26)^  “I was initially kind of shocked that I scored… you know, I thought it would be higher” ^(25)^  “I sort of forgot it… I was a little bit upset when I got it” ^(25)^  “So in what way do you feel I… I’m not doing the right things?” ^(25)^  “Yeh well I eat loads of vegetables and so I found it ah… I am doing things right” ^(25)^  “Now I eat fruit instead of chocolate” (TN 01) ^(24)^  “I know what a good diet is” ^(23)^  “I’m 280 pounds. How can I be malnourished?” ^(23)^  Despite screening tool diagnosing risk of malnutrition, all rated nutritional health as ‘fair’ or ‘good’ ^(23)^  Trying to eat a healthy diet; maintaining a garden to eat fresh fruit and vegetables ^(23)^ | Requests for explanation of final score (meaning of low, medium and high scores) were made ^(19)^ |
|  | Risk perception: lack of understanding of risks and causes of malnutrition and nutrition leading to low risk perception, resulting in low prioritisation of results | “I feel I am not at risk, but I might be wrong” ^(25)^  “I’m 280 pounds. How can I be malnourished?” ^(23)^  “I felt that little applied to me probably because I had not properly completed the questionnaire” ^(25)^  “Well, it doesn’t really bother me” ^(25)^  “I don’t need it. No, we look after ourselves as far as cooking and eating is concerned. I think common sense has got a lot to do with it” (F6) ^(26)^  Cause of weight loss was not cancer, was due to dietary perceived positive dietary changes “I haven’t lost weight because of the therapy, but just because I eat less when I am alone” (TN 02) ^(24)^  “Now I eat fruit instead of chocolate” (TN 01) ^(24)^  “I no longer eat salmon or shrimp or seafood as they can have an effect on the cancer” (TN 08) ^(24)^  “I drank actimel instead of water” (TN 08) ^(24)^  “I will never buy a frozen dinner. There’s no way I’m going to touch that because of the chemicals” ^(25)^  “Well because of the issues I have with my son and his children, I didn’t really take an awful lot of notice of it I’m afraid. I’m sorry, I should have but I didn’t” (F4) ^(26)^ |  |
|  | Understanding role of screening  (low perception of risk, therefore advice is unwanted) | “That was not relevant for me” (TN10) ^(24)^  “I think weight loss is related to everything, food and illness” (TN 06) ^(24)^  “The nurses, they have to ask, that’s what you have to do with all the patients, but that was not relevant for me now” (TN 10) ^(24)^  “Well they can’t do much. It’s me getting old, tired and worried and well, you know (F2) ^(26)^  “I didn’t follow it. No, I didn’t actually – she [practice nurse] told me what cereal to take in the morning but I tried it – one plateful but I couldn’t eat it” (F4) ^(26)^ | Screening seen as unnecessary ^(21)^  Participants understood the need and importance of nutritional screening ^(22)^ |
| **Theme 3**: Barriers to, and opportunities for change – many feel | Rationalising current dietary intake  Lack of readiness to change (lifetime habits) (barriers to change)  Advice does not apply as they are following mainstream guidelines | “That’s what you do with all the patients… but that was not relevant for me” (TN10) ^(24)^  “It’s not a continual practice or we don’t do it for any particular reason other than we’re in a hurry or you’ve had a late breakfast or we’ve been out for breakfast” ^(25)^  “That was not relevant for me” (TN10) ^(24)^  Patients felt comfortable continuing to do things their own way “We saw a dietitian about two or three times. As I said, the advice she gave us, well-meaning, but I didn’t consider it all that helpful (M6) ^(26)^  “I’ve never had soy milk in my life, so I wouldn’t know what it tastes like. And if I don’t know, I wouldn’t buy it” ^(25)^  “You have to have fat on meat to cook it anyway. But see, there is my upbringing” ^(25)^  “I don’t feel I’m as much at risk as… as the community at large. And that’s what bothers me are the people out there. They’re far more at risk I feel” ^(25)^  “Sometimes when you’re working, you’re rushing all the time” ^(25)^  “The recommendations were good for the average person, but like I said, I believe that I eat and watch my diet quite well” ^(25)^  “I’m 280 pounds. How can I be malnourished?” ^(25)^  Participants saw screening results as an assessment of how well they were doing the right things ^(25)^  “Yes, the biggest problem is my appetite. You know, I just don’t feel like eating” ^(25)^  “Well they can’t do much. It’s me getting old, tired and worried and well, you know (F2) ^(26)^  “I am cutting down a little on the amount of red meat we eat but I decided that by myself” (F6) ^(26)^ |  |
|  | Opportunities for learning | “Yes, it was very good that the topic [nutrition] was addressed” (TN09) ^(24)^  “It may be beneficial to all old people I suppose to be quite honest and if things are required after that well it’d most probably be a good thing you know (M2) ^(26)^  “It’s quite informative, very good” (F5) ^(26)^  “It’s a matter of… something we should know about and do something about” ^(25)^  “I count on the medical profession to let me know if they see that there is something wrong. If my weight drops or whatever, then I hope they will ring bells and say “Hey!” ^(25)^  “That’s very important, to try new things. Things that maybe you didn’t grow up with or you just aren’t used to” ^(23)^ |  |
